# Positive Changes in Safety Perception Among Blacks with HIV and Comorbidities: Assessment of Social Determinants of Health During COVID-19

**DOI:** 10.1007/s40615-023-01633-2

**Published:** 2023-05-22

**Authors:** Marc Fleming, Deidra Lee, Chukwuezugo Oranu, Jon C. Schommer, Jennifer Cocohoba, Jennifer Cooper, Crystal K. Hodge, Saharnaz Nedjat, Kathleen Borgmann

**Affiliations:** 1 Department of Pharmaceutical Economics and Policy, Chapman University School of Pharmacy, Harry and Diane Rinker Health Science Campus, 9401 Jeronimo Road, Irvine, CA 92618-1908 USA; 2 AstraZeneca Pharmaceuticals LP, Wilmington, DE USA; 3 Rutgers University, New Brunswick, NJ USA; 4 University of North Texas Health Science, Center College of Pharmacy, Fort Worth, TX USA; 5 Department of Pharmaceutical Care & Health Systems, University of Minnesota College of Pharmacy, Minneapolis, MN USA; 6 Department of Clinical Pharmacy, University of California San Francisco, San Francisco, CA USA; 7 Patient-Centerd Outcomes Research Institute, Washington, DC, USA; 8 University of North Texas Health Science Center System College of Pharmacy, Fort Worth, TX USA; 9 University of Texas Southwestern Medical Center, Dallas, TX USA; 10 Division of Neuroscience & Behavior, National Institute On Drug Abuse, Bethesda, MD USA

**Keywords:** COVID-19, African American, Lockdown, Social determinants of health, Health disparities, Blacks, Minorities, Racial, Ethnic

## Abstract

**Purpose:**

This study aimed to examine the impact of the COVID-19 lockdown on social determinants of health (SDOH) among Blacks with HIV and a comorbid diagnosis of hypertension or type 2 diabetes mellitus (T2DM).

**Methods:**

This was a longitudinal survey study. The inclusion criteria were adults ≥ 18 years and the presence of hypertension and/or diabetes, along with a positive HIV diagnosis. This study enrolled patients in the HIV clinics and chain specialty pharmacies in the Dallas-Fort Worth (DFW) area. A survey of ten questions examining SDOH was conducted before, during, and after the lockdown. A proportional odds mixed effects logistic regression model was applied to assess differences between time points.

**Results:**

A total of 27 participants were included. Respondents felt significantly safer in their living place post-lockdown than in the pre-lockdown period (odds ratio = 6.39, 95% CI [1.08–37.73]). No other statistically significant differences in the responses were found over the study timeframe. However, borderline *p* values indicated better SDOH status post-lockdown as compared to pre-lockdown.

**Conclusion:**

Study participants feel safer one year after lockdown compared to pre-lockdown. The CARES Act and the moratorium on rent and mortgage are among the factors that may explain this increase. Future research should include designing and evaluating interventions for social equity enhancement.

## Introduction

Black populations are disproportionately affected by chronic diseases, including human immunodeficiency virus (HIV)/acquired immune deficiency syndrome (AIDS), hypertension (HTN), and type 2 diabetes mellitus (T2DM). More than 1.1 million people in the US live with HIV/AIDS; among these, 476,100 are Blacks [1, 2]. In 2018, 42% (16,002) of the 37,968 new HIV diagnoses in the US were among Blacks, while they are only 14.2% of the population living in the US [3].

The prevalence of diagnosed diabetes was 8.7% of the US population in 2019, 12.1% among Blacks, compared to approximately 9.5%, 11.8%, 14.5%, and 7.4% among Asians, Hispanics, American Indians, and Whites, respectively [4]. Similarly, 116 million, or almost half of the US population, have HTN. A higher prevalence is seen among historically oppressed race/ethnic groups. HTN is more prevalent in non-Hispanic Black adults (56%) than in non-Hispanic White adults (48%), non-Hispanic Asian adults (46%), or Hispanic adults (39%) [5]. A systematic review of chronic diseases among African Americans found that multiple simultaneous components of health affect African American families. In this study, outcomes related to chronic diseases such as HTN, DM, depression, psychosocial outcomes, and health behaviors were examined [6].

The cause for the health disparities is varied and complex but generally intersects systemic barriers such as economic instability, lack of healthcare access, structural racism, and discrimination [7]. These factors, termed social determinants of health (SDOH), are the primary drivers for health inequity [8]. One of the key elements of SDOH is safe housing, transportation, and neighborhoods [9]. Safety is a basic need and an influential human right, which directly and indirectly affects psychological and physical health [9, 10]. It is defined as protection from physical, social, or emotional harm and is considered a complex concept that various environmental factors and risk management abilities can impact [11].

The coronavirus disease 2019 (COVID-19) pandemic has further strained the healthcare system and exacerbated the burden of chronic diseases. The impact of COVID-19 has been harmful to all individuals; however, it poses an increased risk for individuals with chronic health conditions such as cardiovascular disease, T2DM, obesity, and HIV [12]. Research has shown disparities in COVID-19 outcomes among historically oppressed racial and ethnic populations compared to other groups [13]. A meta-analysis of 68 studies representing over 43 million patients in the United States showed that COVID-19 positivity and severity were higher among African Americans than their White counterparts. Additionally, a positive association between the percentage of the uninsured population and COVID-19 positivity was seen among African Americans [14].

A limited number of studies have examined the effects of non-pharmaceutical interventions (NPIs) such as lockdowns, shelter-in-place orders, and other government-mandated restrictions that limit people’s freedom of movement and activities on COVID-19 mortality. The controversial results of a systematic review concluded that lockdowns failed to affect COVID-19 mortality significantly yet imposed immense economic and social costs [15]. Critics of this paper argue that the conclusions drawn are highly flawed, given the vague characterization of inconclusive efficacy measures for NPIs [16]. These studies set the foundation for our research into how Blacks with chronic diseases were faring regarding the impact of the COVID-19 lockdown on their SDOH. On the other hand, the CARES Act was a stimulus bill passed by the US Congress in response to the economic consequence of the COVID-19 pandemic in the US. Associations between mental health and CARES availability are controversial in different studies [17, 18].

Studies have attempted to characterize the impact of COVID-19 on Black communities. These studies often did not consider individual differences in the severity and positivity of COVID-19 and were conducted at the ecological level; therefore, potential confounders can threaten their validity [19–21]. Our study is uniquely positioned to examine the impact of the lockdown on a particularly vulnerable segment of this community in a longitudinal design at the individual level. Keeping on the same page with Health People 2030 goals related to SDOH, our objectives add to upstream efforts to improve health and reduce disparities [22].

## Methods

### Study Design and Sample

We employed a longitudinal survey as a part of an ongoing clinical trial (NCT03437694). The larger trial aims to assess the impact of medical record-based medication therapy management (MTM) on HIV-related health outcomes. Participants enrolled in the clinical trial were administered a survey and then interviewed. Following this, they received pharmacist-provided MTM services including, but not limited to, prioritization of medication lists, creation of action plans, discussion, and collaboration with a patient and medical provider if applicable, and recommendations for follow-up. The selection criteria for study participation were self-identification as Black, 18 years of age or older, diagnosed with HIV, and had HTN, T2DM, or both as comorbid conditions, as verified by their medical records.

A convenient sample of HIV clinic-recruited participants through multiple recruitment methods in Dallas-Fort Worth (DFW) area was obtained. Once enrolled in the study, participants were scheduled to meet with a pharmacist at a chain specialty pharmacy in the DFW area. For the data presented herein, participants had to have attended at least three study visits (in-person or virtual) from December 22, 2019, to June 22, 2021. This 1.75-year timeframe was subdivided into three periods, and every participant included in this analysis had at least one survey completed within each period. The first period, described here as the pre-lockdown period, was from December 22, 2019, to March 21, 2020. The second period, during the lockdown, heralded by the beginning of the Texas state-wide COVID pandemic restrictions, lasted for three months, from March 22, 2020, to June 23, 2020. One year after locking down, the third period began from March 21, 2021, to June 22, 2021. The study coordinator was present on all visits. He provided medical records to the pharmacist when the patient was in the active treatment arm and conducted the intake survey before the MTM session. The data reported herein were obtained from the intake surveys conducted by the study coordinator.

### Survey Instrument

The original survey instrument comprised 43 questions of a quantitative assessment of barriers to care, health literacy, and medication adherence, among others. These items were developed from the literature review and adapted to address the objectives of the MTM clinical trial. It was a condensed version of the Medical Outcomes Study Scales (MOS) commonly used as a reliable tool in HIV populations [23].

In this study, the analysis was conducted on ten questions from the original survey questionnaire. Based on expert opinions and a review of relevant literature, these ten questions were deemed relevant to SDOH, and there were no additional questions on SDOH in the original questionnaire. All but the final item was measured on a 5-point Likert scale from 1 (strongly agree) to 5 (strongly disagree). The last question asked about the frequency of alcohol consumption in a week. This was also measured on a 5-point scale from 1(very often) to 5 (never). The survey questions addressed the following: whether the participant got enough sleep, whether pain interfered with their work or activities, whether their health interfered with their social activities, whether they spoke to someone they regularly trusted, whether they worried about running out of food, whether they rationed their food due to shortage, whether they had trouble getting their medications, whether their safety was threatened in the place they sleep or live or by someone whom they knew, whether they skipped taking their medications, and whether they consumed over 4–5 alcoholic drinks a day. Responses regarding enough sleep and speaking to someone regularly were reverse-coded for the analytical models and figure.

### Data Analysis

Baseline participant characteristics were described as frequencies and percentages for categorical variables and means and standard deviations (SD) for continuous variables. The distribution of scores for each survey question in each period was evaluated, and medians, interquartile ranges, frequencies, and percentages were reported. To statistically assess whether participants’ responses to the questions changed over time, proportional odds mixed effects logistic regression models were used. Each model included a random participant-level intercept and time as a categorical variable. For each model, the proportional odds assumption was tested and met. Because more than 60% of participants gave the best possible response to each of the last six questions, a secondary analysis was conducted, in which the responses to these questions were dichotomized (5 vs. < 5). We fit binary mixed effects logistic regression models for this analysis, including a random participant-level intercept and time as categorical variables. SAS Enterprise Guide version 8.1 (SAS Institute Inc., Cary, NC) was used for all analyses.

## Results

### Respondent Demographics

Of the 117 participants enrolled to date (8/26/2022) in the trial, 27 participants met the criteria for inclusion in this analysis. Respondents were 52.9 ± 11.0 years of age, mostly female (51.9%), and diagnosed with diabetes and hypertension (51.9%), along with HIV. All patients with T2DM had HTN, too. Respondent characteristics are presented in Table 1.

**Table 1:** Study sample frequency and age categories (*n* = 27)

Characteristics	Frequency, *n* (%)^a^
Age categories	
18–24	–
25–44	7 (25.9)
45–64	18 (66.7)
65 and above	2 (7.4)
Gender	
Male	13 (48.2)
Female	14 (51.9)
Comorbidity	
Hypertension (HTN)	13 (48.2)
Diabetes mellitus & HTN	14 (51.9)

^a^Sum of percentages does not equal 100 due to rounding

### Participant Survey Responses

Table 2 summarizes participant responses to the ten survey items. Generally, most survey respondents disagreed with not having enough food, not getting their medicines because of financial problems, feeling like their safety was threatened in the place they sleep or live, and having to skip medicines for more than one day in the last month. However, respondents were neutral regarding getting enough sleep, pain interfering with work or activities, and health interfering with social activities. Most respondents (> 70%) reported never consuming more than 4 or 5 alcoholic drinks a day within the past week.

**Table 2:** Survey questions and responses at periods pre-lockdown, during the lockdown, and one year after the lockdown

Survey questions				Strongly agree (1)	Agree (2)	Neutral (3)	Disagree (4)	Strongly disagree (5)
In the past week		Median	IQR	Frequency, *n* (%)				
I feel like I got enough sleep.^	Pre-lockdown	3.0	3.0	7 (25.9)	6 (22.2)	4 (14.8)	4 (14.8)	6 (22.2)
	During lockdown	3.0	2.0	5 (18.5)	7 (25.9)	4 (14.8)	7 (25.9)	4 (14.8)
	One year later	2.0	3.0	11 (40.7)	5 (18.5)	1 (3.7)	5 (18.5)	5 (18.5)
My pain interfered with my work or activities.^^^	Pre-lockdown	3.0	2.0	4 (14.8)	6 (22.2)	7 (25.9)	5 (18.5)	5 (18.5)
	During lockdown	3.0	2.0	5 (18.5)	7 (25.9)	4 (14.8)	6 (22.2)	5 (18.5)
	1 year later	2.0	4.0	10 (37.0)	4 (14.8)	–	2 (7.4)	11 (40.7)
My health interfered with my social activities (visiting friends, attending church, etc.).^	Pre-lockdown	3.0	2.0	5 (18.5)	7 (25.9)	3 (11.1)	6 (22.2)	6 (22.2)
	During lockdown	3.0	2.0	4 (14.8)	5 (18.5)	6 (22.2)	7 (25.9)	5 (18.5)
	One year later	4.0	3.0	6 (22.2)	5 (18.5)	2 (7.4)	2 (7.4)	12 (44.4)
I speak with someone I trust regularly	Pre-lockdown	2.0	2.0	13 (48.2)	4 (14.8)	6 (22.2)	1 (3.7)	3 (11.1)
	During lockdown	2.0	2.0	11 (40.7)	6 (22.2)	4 (14.8)	3 (11.1)	3 (11.1)
	One year later	1.0	1.0	15 (55.6)	8 (29.6)	1 (3.7)	1 (3.7)	2 (7.4)
In the past month				Frequency, *n* (%)				
I cut the size or number of meals because I did not have enough food	Pre-lockdown	5.0	1.0	2 (7.4)	–	1 (3.7)	9 (33.3)	15 (55.6)
	During lockdown	5.0	1.0	1 (3.7)	4 (14.8)	–	6 (22.2)	16 (59.3)
	One year later	5.0	1.0	3 (11.1)	1 (3.7)	1 (3.7)	3 (11.1)	19 (70.4)
I did not get my medicines because of financial problems	Pre-lockdown	5.0	1.0	1 (3.7)	1 (3.7)	3 (11.1)	3 (11.1)	19 (70.4)
	During lockdown	5.0	1.0	1 (3.7)	1 (3.7)	3 (11.1)	6 (22.2)	16 (59.3)
	One year later	5.0	0.0	–	2 (7.4)	–	2 (7.4)	23 (85.2)
I felt like my safety was threatened by someone I knew	Pre-lockdown	5.0	0.0	–	1 (3.7)	3 (11.1)	1 (3.7)	22 (81.5)
	During lockdown	5.0	0.0	–	–	–	4 (14.8)	23 (85.2)
	One year later	5.0	0.0	–	–	–	1 (3.7)	26 (96.3)
I felt like my safety was threatened by the place I sleep or live	Pre-lockdown	5.0	1.0	1 (3.7)	3 (11.1)	2 (7.4)	2 (7.4)	19 (70.4)
	During lockdown	5.0	1.0	2 (7.4)	3 (11.1)	–	2 (7.4)	20 (74.1)
	One year later	5.0	0.0	1 (3.7)	1 (3.7)	–	1 (3.7)	24 (88.9)
Since the last study visit, I skipped taking my medicines for more than one day	Pre-lockdown	5.0	1.0	–	3 (11.1)	3 (11.1)	5 (18.5)	16 (59.3)
	During lockdown	5.0	1.0	–	2 (7.4)	1 (3.7)	5 (18.5)	19 (70.4)
	One year later	5.0	0.0	–	2 (7.4)	–	3 (11.1)	22 (81.5)
				Very often (1)	Often (2)	Moderately (3)	Sometimes (4)	Never (5)
				Frequency, *n* (%)				
In the past week, how often did you have more than 4 or 5 alcoholic drinks a day?	Pre-lockdown	5.0	1.0	–	–	1 (3.7)	7 (25.9)	19 (70.4)
	During lockdown	5.0	1.0	–	–	1 (3.7)	6 (22.2)	20 (74.1)
	One year later	5.0	0.0	–	–	–	–	27 (100)

^Sum of percentages may not equal 100 due to rounding

### Responses over Time

**Fig. 1:**
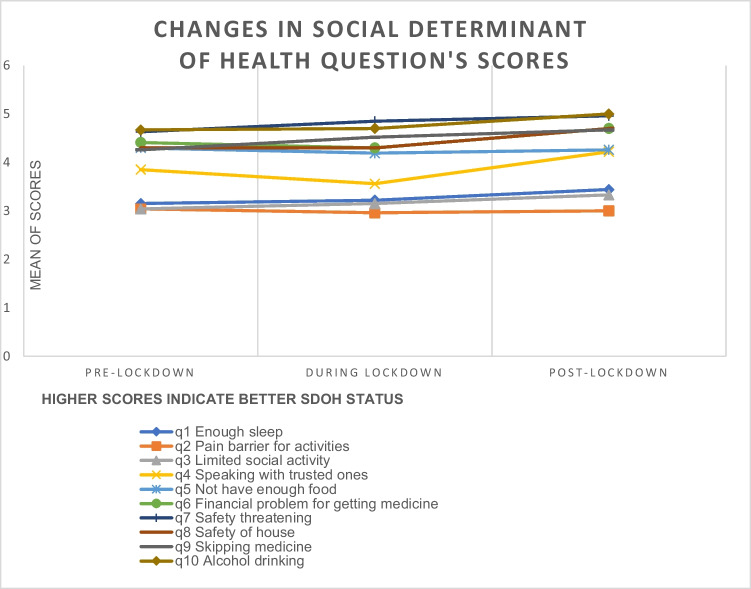
Changes in social determinant of health question’s scores

The proportional odds mixed effects logistic regression model results showed the odds ratios comparing during and one year after lockdown to the pre-lockdown time point (Table 3). A significant difference was found between one year after lockdown and pre-lockdown periods [OR = 6.39, 95% CI (1.08–37.73), *p* = 0.04] for responses to the statement “In the last month, I felt like my safety was threatened by the place I sleep or live” after dichotomization of these responses (strongly disagree vs. all others). In other words, a significantly greater proportion of participants reported a “strongly disagree” response on this question during the post-lockdown period compared to the pre-lockdown period. No other statistically significant differences in the responses were found over the study timeframe. As shown in Table 3, *p* values of odds ratios for the following questions were borderline and showed positive changes when comparing post-lockdown to pre-lockdown time points; “I skipped taking my medicines for more than one day”, “I felt like my safety was threatened by someone I knew”, and “I did not get my medicines because of financial problems”. As depicted in Fig. 1, there were positive changes in response to most survey questions during the study period. However, these changes were not often statistically significant.

**Table 3:** Odds ratios comparing during the lockdown and one year after lockdown periods to the pre-lockdown period

Survey questions		Odds ratio (95% CI) vs. pre-lockdown	*p* value
In the past week		*Proportional odds mixed effects logistic regression models*	
I feel like I got enough sleep.^#^	Pre-lockdown	–	–
	During lockdown	0.83 (0.33–2.04)	0.67
	1 year later	0.66 (0.26–1.71)	0.39
My pain interfered with my work or activities	Pre-lockdown	–	–
	During lockdown	1.26 (0.49–3.28)	0.63
	1 year later	1.09 (0.39–3.04)	0.87
My health interfered with my social activities (visiting friends, attending church, etc.)	Pre-lockdown	–	–
	During lockdown	0.89 (0.35–2.25)	0.8
	1 year later	0.67 (0.25–1.79)	0.41
I speak with someone I regularly trust ^#^	Pre-lockdown	–	–
	During lockdown	1.20 (0.44–3.30)	0.72
	1 year later	0.50 (0.18–1.40)	0.18
In the past month		Binary mixed effects logistic regression models^*^	
I cut the size or number of meals because I did not have enough food	Pre-lockdown	–	–
	During lockdown	1.52 (0.42–5.50)	0.52
	1 year later	3.14 (0.80–12.27)	0.1
I did not get my medicines because of financial problems	Pre-lockdown	–	–
	During lockdown	0.64 (0.17–2.48)	0.52
	1 year later	4.25 (0.84–21.36)	0.08
I felt like my safety was threatened by someone I knew	Pre-lockdown	–	–
	During lockdown	1.96 (0.34–11.33)	0.45
	1 year later	15.60 (0.97–249.88)	0.05
I felt like my safety was threatened by the place I sleep or live	Pre-lockdown	–	–
	During lockdown	1.60 (0.40–6.46)	0.5
	1 year later	**6.39 (1.08–37.73)**	**0.04**
Since the last study visit, I skipped taking my medicines for more than one day	Pre-lockdown	–	–
	During lockdown	2.19 (0.69–6.95)	0.18
	1 year later	2.80 (0.85–9.25)	0.09
In the past week, how often did you have more than 4 or 5 alcoholic drinks a day?	Pre-lockdown	–	–
	During lockdown	1.00 (0.20–5.10)	1
	1 year later	–	–

^#^Reverse coded

^*^ “Strongly disagree” or “never” vs. all other responses odds ratios

“Strongly disagree” or “never” corresponds with a positive attitude or behavior

Values in bold indicate *p*<0.05

## Discussion

A significantly greater proportion of participants reported a “strongly disagree” response to the question “In the last month, I felt like my safety was threatened because of the place I sleep or live” during the post-lockdown period compared to the pre-lockdown period. In other words, patients felt less threatened in their living place post-lockdown compared to the pre-lockdown period. Additionally, despite a small sample size, there was still evidence of positive changes comparing post to pre-lockdown periods for the statements on being threatened by someone they knew, skipping taking medicine because of financial problems, and skipping taking medication more than one day. The significant positive change in feelings of safety in the vulnerable study participants and even the lack of negative changes in other SDOH question responses can be attributed to several factors. Among them, COVID-19-related economic policies like the CARES Act and the moratorium on rent and mortgage could play an important role. The Families First Coronavirus Response Act (FFCRA), CARES Act, and American Rescue Plan (ARP) Act were policies enacted by Congress to provide economic relief from COVID-19 [21]. These policies created moratoriums on foreclosures and evictions to prevent individuals from losing their homes during the pandemic and increase compliance with stay-at-home orders. Economic Impact Payments (EIPs) were also established to provide stimulus payments to low- and middle-income earners [24]. By implementing these policies, the basic needs of the community might be met, and the economic balance in society might be improved. This would reduce economic anxiety, which in turn would decrease instances of domestic violence and other crimes and raise safety levels. However, this hypothesis should be evaluated in future studies, as the studies that assessed the impact of the CARES Act on crime and violence rates have shown inconsistent results [25, 26]. These policies could also improve lifestyle management, including regular medication use.

Despite federal provisions, there is skepticism surrounding whether these policy enactments adequately protected populations as initially designed [27]. A recent review asserted that low-income and historically oppressed racial and ethnic minority communities were disproportionately adversely affected by the pandemic and less likely to receive assistance. Structural inequities, including racism and capitalism, pervasive in our society were partly blamed for this occurrence [20]. It has been shown that these minorities were more likely to be significantly disrupted by COVID-19, possibly due to obstacles to receiving government relief packages [28]. The COVID-19 pandemic has shown social, racial, and economic health disparities and has accentuated effects on vulnerable populations. While we did not include an economic status assessment, our participant population generally represents a marginalized and particularly vulnerable population, especially during the COVID-19 pandemic. A systematic review of the association of racial/ethnic and socioeconomic status (SES) with health outcomes and access to healthcare services during the COVID-19 pandemic found that historically oppressed racial/ethnic minority groups had higher risks of COVID-19 infection and confirmed diagnosis, hospitalization, and death [19]. Factors such as low education level, poverty, poor housing conditions, low household income, speaking a language other than the national language in a country, and overcrowded households were cited as contributing factors [19]. Further, due to safety and effectiveness concerns, COVID-19 vaccination refusal is higher among Blacks than Whites [29].

The Census Bureau’s data revealed an assessment of the SDOH for millions of citizens in the United States during the pandemic. Approximately 7% of adults were not confident in their ability to pay for next month’s housing expenses, and 10.3% reported food insufficiency. Notable disparities exist among these measures for Black and Hispanic adults compared to White adults. As compared to 55.5% of White adults, approximately 75% of Black (74.4%) and Hispanic (75.2%) adults reported difficulty paying their household expenditures in early March 2022, but this survey did not provide pre-pandemic measures for comparison, and its finding showed that changes in SDOH did not follow economic indicators or pandemic trends; therefore, caution is warranted to consider the findings related to the COVID-19 pandemic [30]. Although most studies agree that Blacks and other historically oppressed racial/ethnic minorities in the US suffer the most from the COVID-19 pandemic, studies differ in terminology, outcome measures, inaccurate or incomplete race/ethnicity, and SES data [20].

To our knowledge, no studies have assessed housing security pre-, during, and post-lockdown. Studies have, however, associated housing insecurity with poor health status and stress. A study of a nationally representative sample of US adults reported an association between housing insecurity, higher psychological distress, and lower self-rated health during the COVID-19 pandemic. They also asserted that the CDC’s nationwide eviction moratorium may have mitigated these associations [31]. A study revealed that the CARES Act significantly decreased eviction rates across the US [32]. Therefore, the likely rationale for our findings over the study time includes policy measures enacted to mitigate the economic, social, and health issues, particularly those of low-income and racial/ethnic minority populations. Financial payments and mortgage and rent moratoriums may have contributed to perceived food, income, and housing security.

## Limitations

This study has some limitations that may limit the generalizability of the findings. The major limitation of this study was the use of a regional survey sample for a specifically marginalized population as a nationally representative sample. The restricted geographic location of Dallas-Fort Worth, Texas, as well as the recruitment strategies for this sample, limits the generalizability of the study findings. Also, no measures of SES (income or education level) were collected; as such, no assumptions could be made regarding the respondents’ socioeconomic status baseline to infer any change. A small study sample size should also be considered as one of the reasons for not having statistically significant changes for some questions in this study. Besides, being a subset of a larger ongoing study created limitations related to research design and capabilities. The larger study was not designed specifically to answer the current research question; thus, there is the risk of selection and information biases. Moreover, given data collection in person or via phone, social desirability bias may have influenced participants to respond more positively. Many studies acknowledge social desirability bias as a limitation in interviews and surveys [33–35]. Additionally, the survey instrument used in this study was not validated for this population to address SDOH specifically.

## Conclusions

The participants felt significantly safer where they slept or lived one year after lockdown than pre-lockdown. This change can be attributed to multiple social factors. One of the plausible explanations can be rent and mortgage moratoriums and housing protections provided by the CARES Act. Future research on a representative and large population size is recommended. Evaluating the CARES effect on health disparities will lead to improved awareness that drives policy and practice as we strive to narrow the gaps in health inequity. A multi-pronged approach to addressing the interconnected issues plaguing the healthcare sector and other social and economic sectors will be integral to closing this gap.
